# The complete chloroplast genome of *Bupleurum euphorbioides,* a traditional medicinal plant

**DOI:** 10.1080/23802359.2021.1907802

**Published:** 2021-04-05

**Authors:** Seong-Sik Park, Ji-Hun Jang, Kyung-Min Lee, Seo-Young Lim, Jae-Wan Seo, Sun-Ra Kim, Ho-Kyung Jung, Hyun-Woo Cho

**Affiliations:** Tradition Korean Medicine Research Team, National Development Institute of Korean Medicine, Jangheung, South Korea

**Keywords:** Chloroplast genome, *Bupleurum euphorbioides*, phylogenetic tree analysis, traditional medicinal plant

## Abstract

*Bupleurum euphorbioides* is a rare native plant attributed with analgesic, gallbladder-supportive, and other functions in China and the Republic of Korea. However, the complete chloroplast genome sequence of the native plant *B. euphorbioides* has not been determined. In this study, we sequenced the complete chloroplast genome sequence, and examined the molecular phylogeny and genetic information of *B. euphorbioides*. The total chloroplast genome of *B. euphorbioides* was 154,871 bp in length with a large single-copy region (85,089 bp), small single-copy region (17,714 bp), and pair of inverted repeats regions (26,034 bp). The chloroplast genome encoded a total of 176 genes, including 131 protein-coding genes, 37 tRNA genes, and eight rRNA genes. The phylogenetic tree indicated that *B. euphorbioides* was the most closely related to *B. latissimum.*

*Bupleurum* spp. is one of the largest genera in family Apiaceae, with more than 150 species distributed mainly in Eurasia (Pimenov and Leonov 1993; Neves and Watson 2004). *Bupleurum* species are well-known for their analgesic, antipyretic, gallbladder-supportive, and other functions (Luo and Jin 1991). Therefore, they are very popular in traditional Chinese and Korean medicine (Luo and Jin 1991; Pan 2006; Tan et al. 2007). In the Republic of Korea, native *Bupleurum* species have been analyzed for polymorphic DNA by sequencing (Moon et al. 2009). Of these, *B. euphorbioides* has been designated as a rare plant in 1997 by the Republic of Korea Forest Service. *B. euphorbioides* is a native plant inhabiting the Mt. Seorak, Mt. Sobaek, and Mt. South Deogyu areas (So et al. 2006). However, the complete chloroplast genome of *B. euphorbioides*, native to the Republic of Korea, has not been sequenced.

In this study, we sequenced the complete chloroplast sequence of *B. euphorbioides* and determined its molecular phylogeny and genetic information. Fresh plants of *B. euphorbioides* were collected from Mt. Seorak, Kangwon-do, Republic of Korea (38°11′9″ N, 128°28′54″ E). A voucher specimen (TKMII-33-2) was deposited at the Medicinal Crops Seed Supply Center of the National Institute for Korean Medicine Development (NIKOM). Whole chloroplast DNA was isolated using the DNeasy Plant mini kit (Qiagen, Hilden, Germany), and the raw read sequence (9,377,516 bp) was obtained using the Illumina platform (HiSeq 2500 and NovaSeq) at Genotech Inc. (Yuseong-gu, Daejeon, Republic of Korea). Raw reads having 95% ≥ Q30 (base Phred quality score) were assembled using NOVOPlasty v2.6.7 (Dierckxsens et al. 2017). Raw sequencing data were registered in SRA with accession number SRX9695268. The assembled sequences were annotated using the dual organellar genome annotator (Dogma; Wyman et al. 2004) followed by visualization, analysis, chloroplast genome annotation, and GenBank submission using the tool CPGAVAS2 (Shi et al. 2019). The annotated chloroplast genome sequence was submitted to the NCBI GenBank database under the accession number MT821948. *Bupleurum falcatum* was used as reference genome in all processes.

The chloroplast genome of *B. euphorbioides* included two single-copy regions (large single-copy (LSC) and small single-copy (SSC)) and a pair of inverted repeat (IR) regions comprising 85,089 bp, 17,714 bp, and 26,034 bp, respectively. The chloroplast genome encoded a total of 176 genes, including 131 protein-coding genes, 37 tRNA genes, and eight rRNA genes. The GC content, total LSC, total SSC, and total IR regions constituted 37.7%, 35.8%, 31.4%, and 42.9% of the chloroplast genome, respectively.

For phylogeny of *B. euphorbioides*, 10 complete chloroplast genome sequences belonging to Apiaceae(family) were aligned by MAFFT (Katoh and Standley 2013). Phylogenetic tree was designed by Maximum likelihood(ML) and Bayesian inference(BI) models and constructed via RAxML 8.2.0 (Stamatakis 2014) and MrBayes 3.2.7 (Huelsenbeck and Ronquist 2001), respectively. As a result, based on the resulting phylogenetic tree, *B. euphorbioides* was the most closely related to *B. latissimum* (Figure 1). So, this analysis result could improve understanding for *B. euphorbioides* and provide essential data in the evolution of related groups.

**Figure 1.**
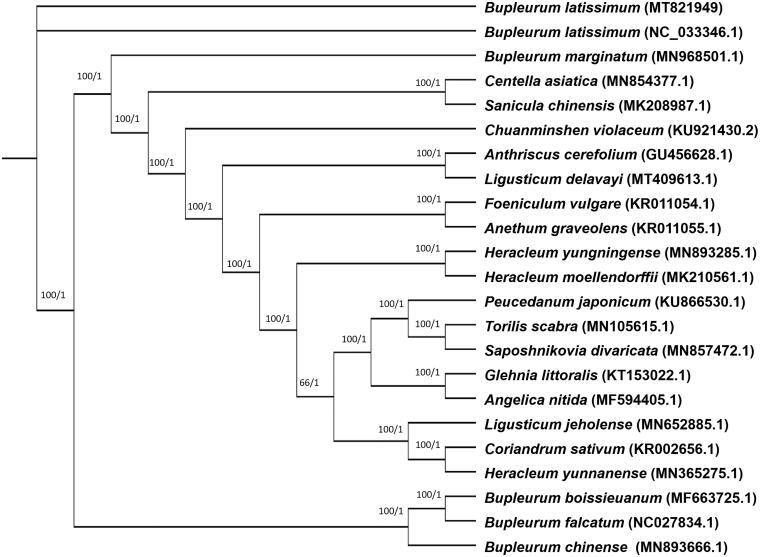
The ML/BI tree inferred from 22 plastid genomes of Apiaceae family. The number on the right is the bootstrap percentage of the ML model and the number on the left is the Posterior provisions of the BI model.

## Data Availability

The genome sequence data that support the findings of this study are openly available in GenBank of NCBI at (https://www.ncbi.nlm.nih.gov/) under the accession no. MT821948. The associated BioProject, SRA, and Bio-Sample numbers are PRJNA685933, SRX9695268, and SAMN17101188, respectively.
